# Visualization of the effect of additives on the nanostructures of individual bio-inspired calcite crystals†
†Electronic supplementary information (ESI) available. See DOI: 10.1039/c8sc03733g


**DOI:** 10.1039/c8sc03733g

**Published:** 2018-11-09

**Authors:** Johannes Ihli, Jesse N. Clark, Nasima Kanwal, Yi-Yeoun Kim, Mark A. Holden, Ross J. Harder, Chiu C. Tang, Sharon E. Ashbrook, Ian K. Robinson, Fiona C. Meldrum

**Affiliations:** a School of Chemistry , University of Leeds , Leeds LS2 9JT , UK . Email: Johannes.Ihli@materials.ox.ac.uk ; Email: F.Meldrum@leeds.ac.uk; b Stanford PULSE Institute , SLAC National Accelerator , Menlo Park , California 94025 , USA; c School of Chemistry and EaStCHEM , University of St. Andrews , North Haugh , St. Andrews , KY16 9ST , UK; d Advanced Photon Source , Argonne , Illinois 60439 , USA; e Diamond Light Source , Harwell Science and Innovation Campus , Didcot , Oxfordshire OX11 0DE , UK; f London Centre for Nanotechnology , University College London , London WC1H 0AH , UK; g Condensed Matter Physics and Materials Science , Brookhaven National Lab. Upton , NY 11973-5000 , USA

## Abstract

Soluble additives provide a versatile strategy for controlling crystallization processes, enabling selection of properties including crystal sizes, morphologies, and structures. The additive species can also be incorporated within the crystal lattice, leading for example to enhanced mechanical properties. However, while many techniques are available for analyzing particle shape and structure, it remains challenging to characterize the structural inhomogeneities and defects introduced into individual crystals by these additives, where these govern many important material properties. Here, we exploit Bragg coherent diffraction imaging to visualize the effects of soluble additives on the internal structures of individual crystals on the nanoscale. Investigation of bio-inspired calcite crystals grown in the presence of lysine or magnesium ions reveals that while a single dislocation is observed in calcite crystals grown in the presence of lysine, magnesium ions generate complex strain patterns. Indeed, in addition to the expected homogeneous solid solution of Mg ions in the calcite lattice, we observe two zones comprising alternating lattice contractions and relaxation, where comparable alternating layers of high magnesium calcite have been observed in many magnesium calcite biominerals. Such insight into the structures of nanocomposite crystals will ultimately enable us to understand and control their properties.

## Abstract 2

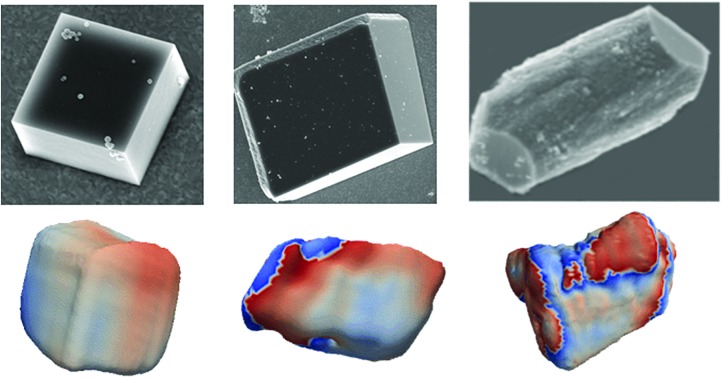
Bragg coherent diffraction imaging to visualize the effects of lysine and magnesium on the internal structures of calcite crystals.

## Introduction

Soluble additives are frequently used in the precipitation of crystalline materials to control properties including the particle size, morphology, and even polymorph.^1^–^3^ There is also growing recognition that this process can be accompanied by the occlusion of the additives within the crystal lattice,^4^–^7^ where this provides an attractive means of tuning the properties of functional materials. Indeed, this has been used to introduce color^8^–^11^ and magnetism,^12^,^13^ enhancing mechanical properties,^14^–^16^ and changing the optical properties and bandgap of semiconductors.^4^,^17^,^18^ Quantum dots have also been incorporated into a range of single crystals as a route to producing stable, and highly luminescent nanocomposites,^19^–^21^ and introducing additional functionality to metal organic frameworks (MOFs).^22^ One of the best examples of the potential of this strategy is provided by the formation of biominerals such as the calcite prisms in mollusc shells^23^–^25^ and echinoid skeletal elements.^26^,^27^ Additives including magnesium ions and organic macromolecules modulate the crystallisation process, resulting in modified morphologies and occlusion of the additives within the crystal lattice.^28^,^29^ The result is single crystals with a substantial increase in hardness and fracture resistance when compared to synthetic calcite.^15^,^30^–^32^

In order to fully exploit soluble additives to control crystal properties, we need a detailed understanding of their influence on the internal structures of individual crystals, with nanoscale resolution. While techniques such as powder X-ray diffraction (P-XRD) and vibrational spectroscopies are ideal for determining the average effect of additives on crystal structures,^33^,^34^ they provide no information on low-frequency inhomogeneities such as defects or compositional zoning. These can arise from the interaction of the additives with the crystal surface during growth and their occlusion in the crystal lattice, and can have a significant impact on the physical properties of crystals. Transmission electron microscopy (TEM) is making significant advances in this regard, where it is now even possible to image the displacement of thousands of individual atoms from their ideal lattice positions in 3D, with picometer resolution.^35^ Yet, electron microscopy is limited to nanoscale objects, and the preparation of thin sections and the imaging process itself can lead to the formation of artefacts.^36^–^38^ While common X-ray microscopy methods also show great potential, they as yet fail in one of the required criteria of spatial-resolution, dimensionality or chemical sensitivity.^39^,^40^

Emerging coherent X-ray diffraction imaging (CDI) methods can potentially overcome these limitations,^25^,^41^,^42^ where these techniques eliminate common X-ray optics and replace them with an iterative phase retrieval algorithm for image generation.^43^ This article describes the use of Bragg Coherent Diffraction Imaging (BCDI) to visualize the inhomogeneities present in individual micron-sized calcite crystals precipitated in the presence of organic and inorganic additives with nanoscale resolution. Investigating amino acids and magnesium ions as important additives, we demonstrate that crystals incorporating low levels of lysine (Lys-calcite) and magnesium ions (Mg-calcite) both alter the internal structures of the crystals. While lysine creates dislocations within the Lys-calcite crystals, Mg ions generate multi-component strain profiles. Further, alternating layers of high magnesium calcite are observed that are comparable to those observed in many magnesium calcite biominerals.^26^,^27^ This work provides unique insight into the mechanisms by which additives control material structures and suggests that crystals with specific strain profiles – and thus physical properties – can be generated by judicious selection of additives.

## Results

**Fig. 1:**
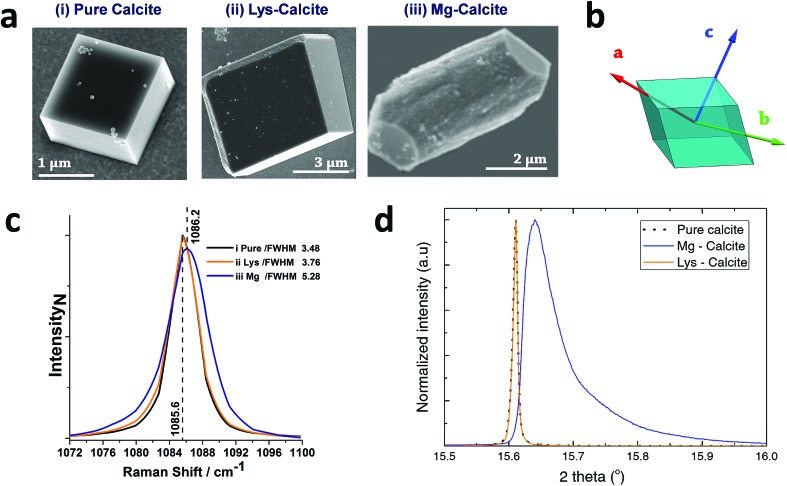
General sample characterization. (a) SEM images of calcite crystals precipitated from a 5 mM CaCl_2_ solution using the ammonia diffusion method (i) pure calcite, (ii) calcite grown in the presence of 2 mM lysine and (iii) calcite grown in the presence of 2 mM magnesium. (b) Calcite crystal showing a rhombohedral morphology bounded by {104} faces, and indicating the crystallographic axes. (c) The 1086 cm^–1^ Raman peak (normalized Intensity) of pure calcite, Lys-calcite and Mg-calcite crystals, emphasizing the effect of these additives on the crystal lattice. Full spectra are presented in the ESI.† (d) HR-PXRD patterns, showing the profiles of {104} reflections of the three types of calcite crystals.

Calcite crystals incorporating either lysine or magnesium ions were precipitated using a vapor diffusion method,^44^ where droplets of a 5 mM CaCl_2_ solution containing 2 mM Lys or 2 mM Mg^2+^ were deposited on hydroxyl-terminated self-assembled monolayers (SAMs). These surfaces support the preferential alignment of the calcite crystals on {104} faces, minimize sample drift during the coherent imaging experiments, and facilitate the experimental alignment. Calcium carbonate precipitation was then induced by exposure to ammonium carbonate vapour for 30 minutes. Crystals were primarily calcite, with a minor fraction of vaterite, as demonstrated by Raman spectroscopy and synchrotron high resolution Powder XRD (HR-PXRD) (Fig. 1). The product crystals were also analysed using scanning electron microscopy (SEM) (Fig. 1a), where pure calcite crystals are rhombohedral in morphology and express six {104} facets. Calcite crystals grown in the presence of lysine (Lys-calcite) were also rhombohedral with very minor edge truncations, while magnesium ions induced a much greater morphological change due to their preferential interaction with the acute step edges. This resulted in magnesian calcite crystals (Mg-calcite) that are elongated along the crystallographic *c*-axis, and capped with {104} facets.^45^,^46^

Analysis of the average compositions of the crystals showed that they comprised ≈1.8 mol% Mg, as determined by atomic absorption, and ≈0.3 mol% Lys, as shown by fluorescence derivatization of the amino acids released on dissolution of the crystals.^47^ Raman spectroscopy was used to assess the influence of these additives on the calcite crystal lattice (Fig. 1c and S1†). The main Raman peak at 1086 cm^–1^, which arises from the symmetric stretch of the carbonate group, shifted from 1085.6 cm^–1^ to 1086.2 cm^–1^ on occlusion of Mg^2+^, where these smaller ions substitute for Ca^2+^ in the lattice. The full width half maximum (FWHM) peak width also increased by 50% as compared to pure calcite, where this can be attributed to a decrease in the coherent scattering domain size, chemical inhomogeneity in the crystal and an increase in dislocation/defect density.^48^ The Lys-calcite crystals, in contrast, showed no shift in the 1086 cm^–1^ peak, and only an 8% increase in the FWHM peak width.

Synchrotron HR-XRD (Fig. 1d) supported these analyses, where Rietveld refinement and line profile analysis of the diffraction patterns revealed virtually no change in the coherent scattering domain size or the lattice parameters for Lys-calcite as compared with pure calcite. In contrast, the diffraction patterns from the Mg-calcite samples revealed a shift and asymmetric broadening in the dominant peak positions (blue arrow) towards smaller *d*-spacings, as exemplified by a change of the {104} Bragg reflection from 0.011° to 0.086° (integral breadth). Analysis of the patterns revealed a reduction in the domain size from 650 nm to 106 nm, and large lattice distortions of Δ*c*/*c* = 0.0028 and Δ*a*/*a* = 0.0017. The higher lattice distortion along the *c*-axis is consistent with the greater elasticity of calcite in this crystallographic direction.^15^ An average occlusion level of 2.1–2.3 mol% Mg within the calcite lattice was calculated using the lattice parameters obtained.^49^

These measurements give information about the average changes in the crystal lattice (P-XRD) and the carbonate ion environment (Raman) on occlusion of these additives. BCDI, in contrast, provides a unique opportunity to determine the local effects of the additives, generating three-dimensional visualizations of the morphology, internal structure, strain fields and associated dislocation network within individual crystals.^50^,^51^ In BCDI, the 3D diffraction pattern corresponding to a selected Bragg peak (here {104}) of an isolated crystal is measured. This is achieved by gradually rocking a coherently illuminated sample through a selected Bragg peak during which a series of 2D diffraction patterns are acquired. Inversion of the 3D diffraction pattern by means of a phase retrieval algorithm yields a complex-valued 3D image of the sample (Fig. S2†). The reconstructed amplitude provides a representation of the electron density distribution in the specimen with nanometre resolution (here ≈100 nm), while phase shifts in the complex amplitude provide a 3D visualization of lattice strains with sub-Ångström sensitivity, *i.e.* the displacement of atoms from the ideal lattice points and scattering vector *Q*.^42^,^52^ Using both types of image contrast, this technique can also be used to visualize selected crystallographic defects.^50^,^51^ Details regarding BCDI experiments, image reconstruction and spatial resolution estimation are provided in the ESI Methods and Fig. S3.†

**Fig. 2:**
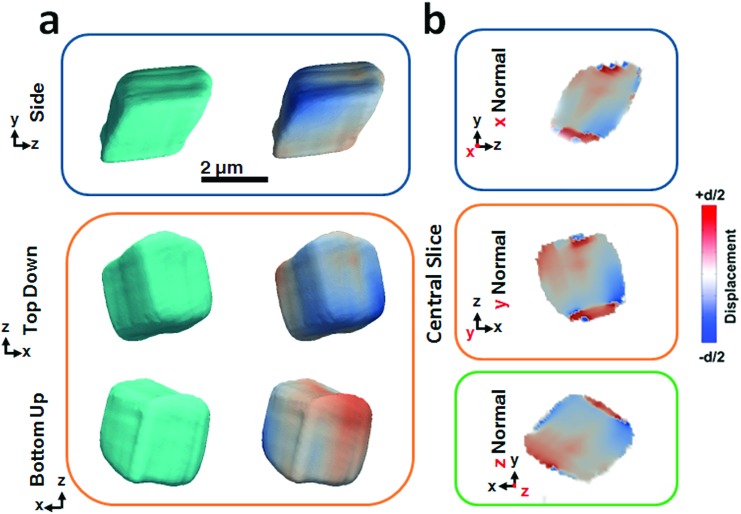
Summary of BCDI reconstructions collected of a pure calcite crystal. (a) Side, top-down and bottom-up projections of the reconstructed crystal shapes from BCDI amplitude measurements and the projected displacements (–*d*/2 blue lattice dilation and +*d*/2 red lattice contraction). (b) Sections through the centre of the crystal of the displacement maps normal to each major axis *x*, *y*, *z*. No dislocations could be identified. The beam direction is along the *z* axis, with the *y* axis vertical, while the sample/substrate is located at a set scattering angle towards the beam direction (*z*).

Fig. 2 shows BCDI image reconstructions of a pure calcite crystal that is ≈2.5 μm in diameter. The reconstructed electron densities (amplitudes) provide a visualization of the crystal morphology (Fig. 2a), where this is comparable to the SEM images shown in Fig. 1a. The projected displacements (phase), in turn, correspond to lattice strains and are represented by a cyclic colour map projected onto the recorded electron density (Fig. 2a). A colour shift towards red (+*d*/2) corresponds to a lattice contraction, while a shift towards blue (–*d*/2) equates to lattice dilation, where *d* is equal to the spacing between two adjacent {104} lattice planes. Sections through the centre of the crystal of the displacement maps normal to each major axis *x*, *y*, *z* are shown in Fig. 2b. The crystal can be seen to experience lattice contractions in the order of ≈0.5 Å perpendicular to the substrate, where these are visible up to 300 nm normal to the substrate. The solution-facing {104} facets experience surface relaxation (ESI Movie 1†). No dislocations could be identified, where these are characterized by the combination of a spiral displacement and a low electron density core.^50^,^51^,^53^

**Fig. 3:**
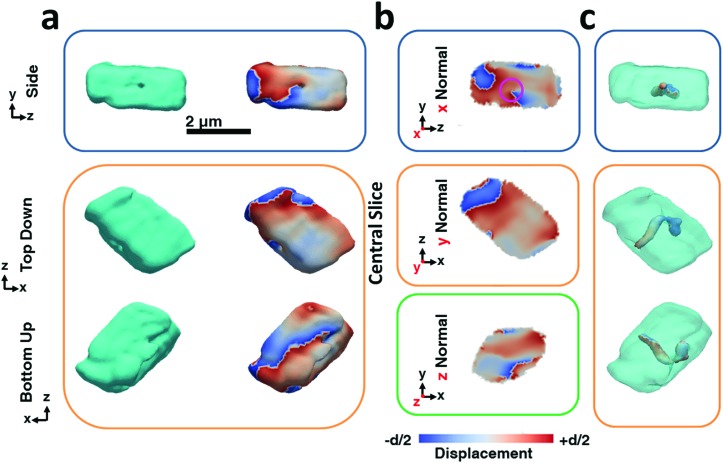
BCDI reconstructions of a calcite crystal grown in the presence of lysine. These crystals incorporate ≈0.3 mol% of lysine. (a) Side, top-down and bottom-up projections of the reconstructed crystal shapes from BCDI amplitude measurements and the projected displacements (–*d*/2 blue lattice dilation and +*d*/2 red lattice contraction). (b) Sections through the centre of the crystal of the displacement maps normal to each major axis *x*, *y*, *z*. (c) shows surface renderings of the defects present within the crystal. Defects are specified in respect to their location within the crystal as given by semi-transparent projections of the electron density. The defects shown have a low electron density core that is surrounded by a spiral deformation field/phase. Areas of spiral displacement (*○*) are highlighted. The beam direction is along the *z* axis, with the *y* axis vertical, while the sample/substrate is located at a set scattering angle towards the beam direction (*z*). The scattering angle is nearly parallel to the *y* axis.

The BCDI reconstructions show that precipitation in the presence of Lys causes marked differences in the calcite crystals (Fig. 3). While the image reconstructions are again in morphological agreement with the SEM images and confirm single crystal character, analysis of the strain within the crystal reveals the presence of a single dislocation. The iso-renderings displayed in Fig. 3c show that this is positioned such that it originates at the SAM/crystal interface and then loops over to terminate on one of the larger side faces. (ESI Movie 2†). The spiral displacement associated with the dislocation is clearly visible in the section shown in Fig. 3b (highlighted with a pink circle) and in the side projection given in Fig. 3a. Interestingly, the atomic displacement associated with the tail of the screw dislocation facing the growth solution is visible over the entirety of the crystal face, which indicates that crystal growth is dominated by this single screw dislocation at this stage of its development. Looking further at the strain, it is seen that the crystal is divided along the dislocation line into a heavily strained top half and a nearly undisturbed bottom half. The top half undergoes multiple phase wraps *i.e.* experiences an accumulated lattice displacement greater than a unit cell from the dislocation line centre towards the crystal faces. ESI Movie 2† further illustrates this, providing slices through the displacement maps parallel and perpendicular to the dislocation line. It is noted, however, that as our data were recorded from the {104} reflection only, we would not necessarily have been able to image any dislocations lying parallel to {104} planes.^50^ Measurements from a further two reflections would be required to sample the full scale tensor.^54^ While successful reconstruction of data from a further crystal also revealed the presence of a single dislocation, we would expect this to vary across a population of crystals such that some may exhibit no, or multiple dislocations. Indeed, the number of dislocations will also vary according to the crystal growth conditions.^50^

**Fig. 4:**
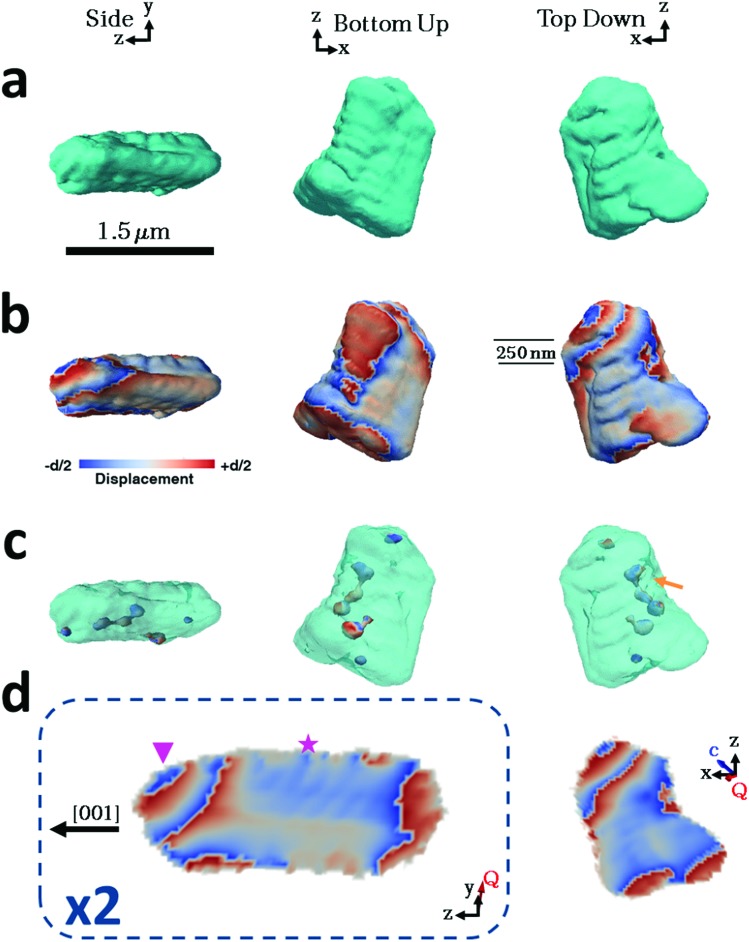
BCDI reconstructions of a calcite crystal grown in the presence of magnesium ions. These crystals incorporate ≈1.8 mol% Mg. Side, top-down and bottom-up projections of the reconstructed crystal shapes from (a) BCDI amplitude measurements and (b) the projected displacements (–*d*/2 blue lattice dilation and +*d*/2 red lattice contraction). (c) Surface renderings of the defects present within the crystal. (d) Sections through the centre of the crystal of the displacement maps normal to the *y* and *x* axes. The star and triangle highlight repeated lattice contraction and relaxation. The beam direction is along the *z* axis, with the *y* axis vertical, while the sample/substrate is located at a set scattering angle towards the beam direction (*z*). The scatting angle *Q* is near parallel to the *y* axis.

Considering then the Mg-calcite crystals, the diffraction patterns used in the construction of the iso-surface renderings of the crystal shown in Fig. 4a were collected at a *d*-spacing of 3.031 Å, as compared to 3.035 Å for pure calcite. Application of Vegard's law^55^ yields a homogenous magnesium content in this particular crystal of ≈1.6 mol%. This is slightly less than the 1.8 mol% obtained as an average of the population of crystals using atomic absorption or 2.1% as determined using HR-PXRD. Unlike the pure calcite and Lys-calcite examined, the iso-surface renderings of the reconstructed electron density do not resemble the crystals observed in the electron microscope. The imaged crystal is significantly smaller (≈1.5 μm) and exhibits a layered structure with curved facets on one side of the crystal when compared to the 4 μm, well-faceted crystals observed by SEM. The corresponding 3D diffraction pattern was also highly distorted. This is not surprising when the increased defect density and the decreased domain size (as determined *via* Raman and P-XRD) are considered.^56^ Parts of the crystal that do not satisfy the selected Bragg condition – such as twin domains or “miss-oriented grains” – will be invisible in the retrieved electron density maps.^53^ A similar discrepancy between the morphology and iso-surface rendering was observed in the reconstruction of a magnesium-rich calcitic prism found in the shell of the mollusc *Pinctada margaritifera* by Bragg ptychography.^25^

The iso-surface renderings of the projected displacements reveal that the layered structure is translated into the atomic displacement (Fig. 4b). The crystal examined additionally possesses one major dislocation that runs approximately parallel to the long axis of the reconstructed crystal, and perpendicular to the layers (Fig. 4c, orange arrow). In the absence of a well-defined crystal morphology, the knowledge that the scattering vector *Q* in the image reconstruction lies nearly parallel to the *y* axis, and that the orientation of a dislocation is governed by the preferential calcite slip system of {104} and {012} planes and axis, and that the orientation of a dislocation is governed by the preferential calcite slip system of {104} and {012} planes and 〈4̄ 2̄ 1〉 slip, can be used to determine that the dislocation lies approximately parallel to a {104} facet and that the layers are preferentially oriented with respect to a growth direction of the calcite crystal (ESI Movie 34[combining macron] 2[combining macron] 1 axis, and that the orientation of a dislocation is governed by the preferential calcite slip system of {104} and {012} planes and 〈4̄ 2̄ 1〉 slip, can be used to determine that the dislocation lies approximately parallel to a {104} facet and that the layers are preferentially oriented with respect to a growth direction of the calcite crystal (ESI Movie 3 slip, can be used to determine that the dislocation lies approximately parallel to a {104} facet and that the layers are preferentially oriented with respect to a growth direction of the calcite crystal (ESI Movie 3†).

Further examination of this repeated lattice contraction and relaxation using slices cut through the centre of the atomic displacement maps (Fig. 4d) reveals that this pattern comprises two components at different length scales. The first comprises localized lattice relaxations (≈50 nm in thickness and 200 nm in length) which are roughly parallel to the long axis of the crystal and run along its whole length. These are marked with a star in Fig. 4d and exhibit average displacements in the *d*-spacing of 20% or ≈0.6 Å. Considering the possibility that these displacements solely arise from compositional differences, this displacement would correspond to a change in the local Mg concentration of 20 mol% from the average value of 1.6 mol%. It is therefore more likely that it arises from a combination of increased point defect density and elemental substitution. The second component is repeated on a larger scale and is tilted at ≈45° with respect to the long axis of the crystal (indicated with a triangle). This appears as a repeated contraction/relaxation of multiple phase wraps spanning the width of the crystal. Again, data were obtained from a further four crystals, of which one was reconstructed and showed comparable structures. The others were too strained to enable successful reconstruction.

**Fig. 5:**
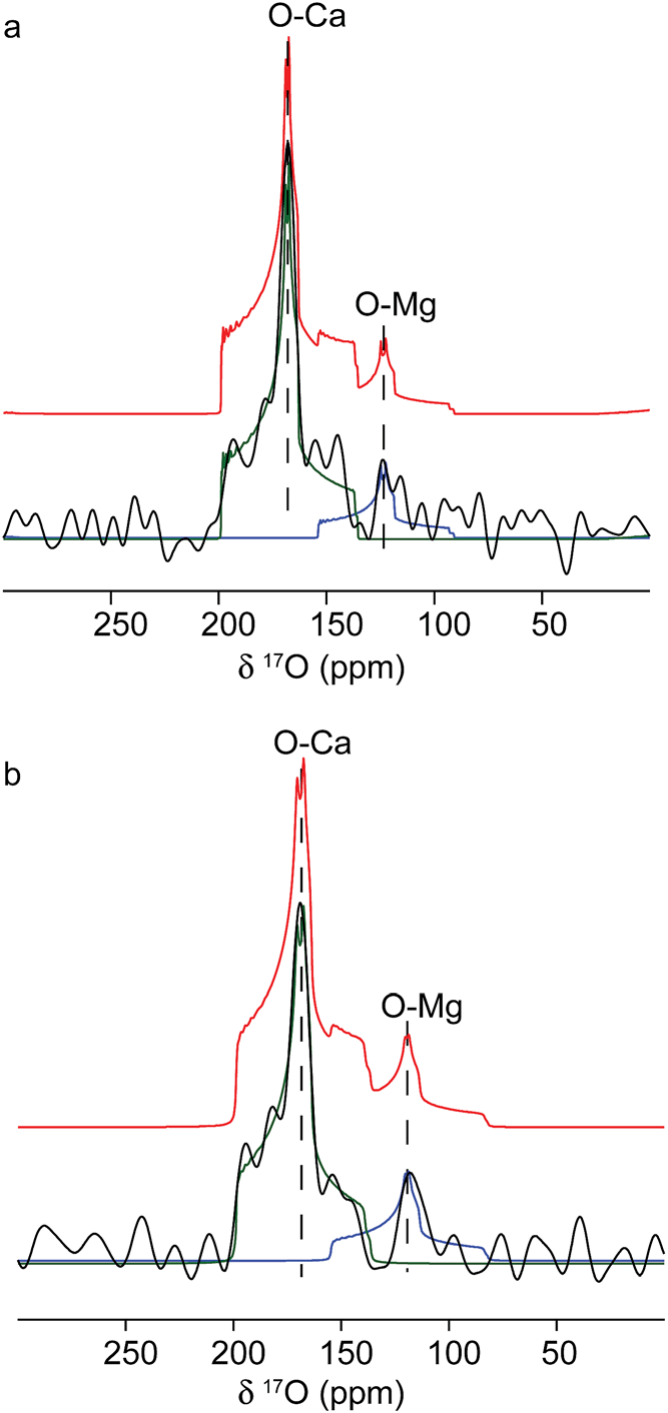
^17^O MAS NMR spectra of calcite crystals precipitated at (a) 10/2 Ca/Mg and (b) 10/4 Ca/Mg, acquired at 20.0 T in a 2.5 mm rotor spinning at 29.5 kHz. Also shown are lineshapes simulated using the NMR parameters (initially obtained from periodic DFT calculations for Mg-substituted calcite with parameters then adjusted to fit the experimental spectrum). Individual lineshapes are shown in blue and green (for O–Mg and O–Ca species, respectively), and the summed lineshape is shown in red.

The origins of these different strain components within the Mg-calcite lattice were then investigated further using ^17^O solid-state NMR spectroscopy^57^,^58^ to determine whether they were associated with different local chemical environments. Conventional Magic-Angle Spinning (MAS) NMR spectra^57^,^59^ were recorded for calcium carbonate samples that had been precipitated by combining solutions of ^17^O-enriched carbonate and calcium/magnesium ions, at calcium/magnesium ratios of 10/2 and 10/4. Fig. 5 shows the ^17^O MAS NMR spectrum of the sample produced at 10/4 Ca/Mg, acquired at 20.0 T. Two distinct resonances centred at about 165 ppm and 120 ppm are observed, with features characteristic of second-order quadrupolar broadening. The spectrum can be fitted with two ^17^O sites with isotropic chemical shifts of 199 ppm and 155 ppm and quadrupolar coupling constants (*C*_Q_) of 7 MHz and 7.5 MHz. These can be attributed to Ca–O and lattice substitutional Mg–O environments, respectively. The fits were obtained by simulating initial powder-pattern lineshapes with NMR parameters from CASTEP density functional theory (DFT) calculations^60^,^61^ on calcite, magnesite and a Mg-substituted calcite model system, and then adjusting the parameters to fit the experimental spectrum. Identical shifts were observed with the sample produced at 10/2 Ca/Mg, although the contribution at lower shift (*i.e.*, the Mg-substituted environments) was less pronounced. While these data provide direct evidence for Mg substitution in calcite, it is not possible to differentiate different types of Mg environment, given the low intensity of the ^17^O signal associated with Mg ions.

## Discussion

**Fig. 6:**
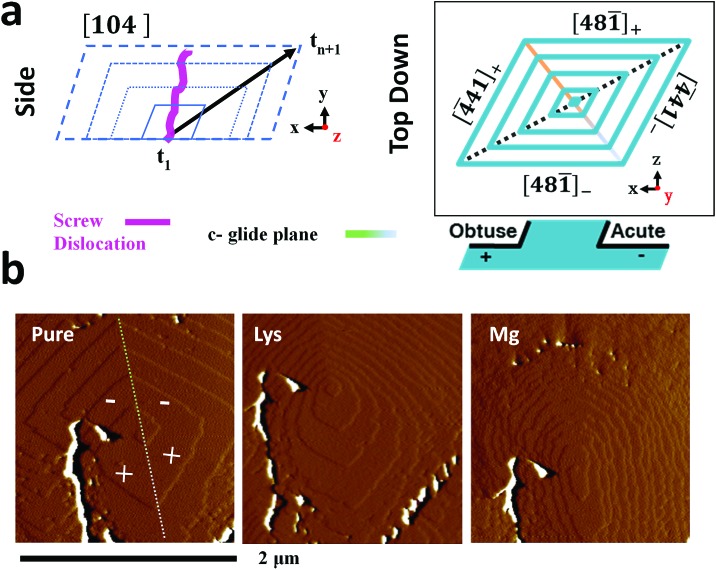
Additive interaction with a calcite single crystal. (a) Schematic of the growth of a calcite crystal, bounded by {104} faces at different time points (*t*), and of the geometry of the steps present on a {104} plane. (b) Atomic force micrographs (deflection) of a growth hillock after overgrowth in a 0.25 mM “CaCO_3_” growth solution without additives (left), in the presence of 0.1 mM lysine (centre) and 0.1 mM magnesium (right). The acute (–) and obtuse (+) steps are indicated. The hillock was overgrown with pure “CaCO_3_” growth solution between additives to establish a common base. The white/black features, predominantly to the left of the hillock, result from much larger deflection of the cantilever due to a relatively deep crack in the crystal surface. This was present on the seed crystal prior to the introduction of the overgrowth solution. It can be used as an “anchor point” to demonstrate that step growth from the same dislocation is imaged in all three examples.

The additive-induced strain profiles observed within the individual calcite crystals provide a unique signature of the interaction between the crystal and the additive, and of the incorporation of the additives within the crystal lattice. Under the low supersaturation conditions employed, crystal growth is dominated by screw dislocations operating on {104} faces (Fig. 6a).^46^,^62^ Due to the symmetry of the calcite lattice, these steps lie at acute (designated [4[combining macron]41]_–_ and [481[combining macron]]_–_) or obtuse (designated [4[combining macron]41]_+_ and [481[combining macron]]_+_) angles to the {104} planes.^63^ As calcite growth is dominated by the availability of kink sites on the step edges,^64^,^65^ additive-directed crystal growth must be considered in terms of molecular recognition at these distinct step edges, where the acute and obtuse step edges each present a unique environment. Smaller ions such as Mg^2+^^66^ and amino acids^62^ preferentially bind at the acute steps, while larger species such as Sr^2+^^67^ often show a preference for the obtuse edges. This inequivalent binding to the step edges then translates into macroscopic changes in the crystal morphologies and variations in the local composition of the crystal.

Evidence of these step-specific interactions can be obtained from atomic force microscopy (AFM). Fig. 6b shows images of a calcite growth hillock after overgrowth in (i) an additive-free, low supersaturation (0.25 mM) calcium carbonate solution, and in the presence of (ii) 0.1 mM Lys, and (iii) 0.1 mM Mg^2+^. Lysine demonstrates a clear preference for the acute steps, where its addition to the growth solution causes a roughening of the two acute steps on the well-faceted hillock, while the obtuse steps remain unaffected. A decrease in terrace width – which is indicative of step pinning – is seen disproportionately for the acute steps. The ratio of terrace widths changes upon addition of lysine from 1_obtuse_ : 1_acute_ to 1_obtuse_ : 0.22_acute_. These microscopic changes in crystal growth cause the truncations of the calcite rhombohedron seen in Fig. 1.

The interaction of magnesium ions with calcite has been the subject of considerable interest.^45^,^46^,^68^–^71^ Like lysine, magnesium ions also preferentially bind to the acute over the obtuse steps. Under the growth conditions employed here, both the acute and the obtuse steps roughen, but the effect on the acute steps is greater, which causes the hillock to adopt an elliptical form. The greatest specificity is typically observed at lower supersaturations, and lower Mg/Ca ratios.^46^,^68^ On a macroscopic scale this results in a pile-up of {104} step edges, and the formation of roughened, pseudo-{*hk*0} faces, as shown in Fig. 1. The retardation of calcite growth by Mg^2+^ ions is typically attributed to the high dehydration energy of these ions,^70^,^72^ and the higher solubility of Mg-calcite.^46^

In this system, the stronger interaction of Mg^2+^ ions with calcite, than Lys with calcite, also translates into higher incorporation levels, with the crystals comprising 1.8 mol% Mg^2+^ and 0.3 mol% Lys. It is noted, however, that this relationship is not universal; additives can exert a strong morphological effect and yet be poorly occluded, while others that exhibit high occlusion efficiencies have little effect on morphology.^15^,^73^ The relationship between the growth conditions and Mg occlusion is complex. There is a strong correlation between the Mg/Ca ratio in solution and Mg occlusion, where this can be independent of,^65^,^72^ or increase with the crystallisation rate,^69^,^74^ depending on the supersaturation regime.

The different occlusion levels in the Lys-calcite and Mg-calcite crystals, together with the changes in the crystal growth that occur in the presence of these additives, necessarily contribute to the contrasting strain profiles present in the crystals. Considering first the Lys-calcite crystals, strain is concentrated along the dislocation line, and then rapidly relaxes away from this. This relaxation is particularly effective along the *c*-axis (where this can be identified from the crystal morphology), as was also observed in the HR-PXRD data. This is readily rationalised based on the anisotropic crystal structure of calcite, where the layers of carbonate ions parallel to the *c*-axis render it more elastic along this direction.^33^,^34^

Looking then at the Mg-calcite crystals, magnesium readily incorporates into the calcite lattice.^45^ The majority of the magnesium is homogenously distributed throughout the crystal lattice as a solid solution, which generates the macrostrain (average lattice distortions) observed in PXRD. As this strain is homogeneous, it is not observed in the BCDI image reconstructions. The inhomogeneous strains observed are instead indicative of local variations in the elemental composition in the crystal. A range of phenomena can give rise to this effect. Geological and synthetic calcite crystals occluding Mg^2+^ ions often exhibit intra-sectorial zoning,^67^ where only a sub-volume of a single growth sector (that associated with symmetry-related crystal faces) takes up the additive. Preferential association with the acute step edges results in higher Mg concentrations in the equatorial zone of the crystal. This cannot be seen here due to the inhomogeneous strains present within the crystal, which dominate the strain profile. Magnesium ions are also known to accumulate in nano-domains in high-Mg biological calcite crystals, as observed using high resolution transmission electron microscopy (HR-TEM),^26^ electron diffraction^27^ and Bragg ptychography,^25^ and in geological calcite crystals, as seen using X-ray absorption fine structure (XAFS).^75^ The strain profile in our crystals gives no evidence for this phenomenon, which is expected based on their low Mg contents.

Considering the two types of inhomogeneous strains observed, the first (marked with a triangle in Fig. 4d) appears as micron-spaced phase wraps across the width of the crystal. The tilt angles of the planes containing the phase wraps lie at 40–50° to the *c*-axis of the crystal (its long axis),^46^ such that the layers of equal displacement lie approximately parallel to a {104} face (which lies at 45° to the *c*-axis). These can be attributed to oscillatory zoning, which occurs due to local fluctuations in the solution composition, and gives rise to zones of higher/lower impurity concentrations and defect density at specific time-points in the evolution of a crystal.^76^ Such zones of equal composition therefore lie in a plane parallel to the crystal growth front. Interestingly, similar alternating layers of high magnesium calcite have previously been observed in magnesium calcite biominerals.^24^,^25^,^77^

The second feature (starred in Fig. 4d) comprises localized lattice relaxations, where zones of equal displacement lie approximately perpendicular to the long axis of the crystal. These cannot be attributed to oscillatory zoning as the strains observed are maintained throughout crystal growth. A possible explanation for this effect can be found in the so-called “template effect” in which topographic features present on the crystal surface are reproduced in subsequent lattice layers.^70^ This has been observed in AFM studies of the growth of magnesium calcite on pure calcite,^70^ where it was suggested that incorporation of magnesium ions gives rise to a local strain profile which then influences the occlusion of further foreign ions in subsequent layers. Following the initial random occlusion of magnesium ions at early stages of growth, where lower levels of occlusion are expected,^78^ more concentrated domains may therefore propagate during subsequent crystal growth.

## Conclusions

In summary, this work demonstrates that BCDI offers a unique tool for visualizing the internal structures of individual calcite crystals grown in the presence of soluble additives. It therefore complements common methods such as P-XRD and vibrational spectroscopies that provide data averaged over a population of crystals. BCDI of a calcite crystal precipitated in the presence of lysine revealed that the amino acid was uniformly distributed throughout the crystal, and that its interaction with the growing crystal resulted in the formation of a single screw dislocation that dominated crystal growth. The magnesium ions, in contrast, exerted a much stronger effect on the calcite crystal lattice. In addition to a homogenous solid solution of magnesium ions in the calcite lattice, two distinct inhomogeneous strain features were also present, where these arise from zones of high magnesium calcite. Magnesium ions are therefore expected to have a much greater effect on the mechanical properties of calcite than amino acids, as is observed experimentally. Future work will determine how such crystal dopants control mechanical properties, where this will be achieved using *in situ* measurements and anomalous scattering effects to determine the individual contributions of elemental and structural inhomogeneities.

## Conflicts of interest

There are no conflicts to declare.

## Supplementary Material

Supplementary information

Supplementary movie

Supplementary movie

Supplementary movie
