# Supplementary material for: Visualization of the effect of additives on the nanostructures of individual bio-inspired calcite crystals
Source: Chem Sci. 2018 Nov 9;10(4):1176–85. doi: 10.1039/c8sc03733g (PMC6349071; doi:10.1039/c8sc03733g)
Supplement: Supplementary file 1 [file SC-010-C8SC03733G-s001.pdf]

Electronic Supplementary Material (ESI) for Chemical Science.
This journal is © The Royal Society of Chemistry 2018

Supplementary Information

Visualization of the Effect of Additives on the Nanostructures of Individual Bio-Inspired Calcite Crystals

Johannes Ihli, Jesse N. Clark, Nasima Kanwal, Yi-Yeoun Kim, Mark A. Holden, Ross J. Harder, Chiu C. Tang, Sharon E. Ashbrook, Ian K. Robinson and Fiona C. Meldrum

1. Materials and Methods

Materials. Analytical grade $(NH_4)_2CO_3$, $CaCl_2$:$2H_2O$, $MgCl_2$:$6H_2O$, L-Lysine were purchased from Sigma-Aldrich and were used as received. $H_2{}^{17}O$ (10%) was purchased from Cortecnet. Aqueous solutions were prepared using Milli-Q Standard 18.2 MΩcm and experiments were performed at a temperature of 21°C. Glassware was soaked overnight in 10% w/v NaOH, followed by rinsing with dilute HCl and finally washing with Milli-Q water.

Self-assembled monolayers (SAMs). SAMs were prepared on freshly-deposited Cr/Au films supported on silicon wafers or glass slides. The substrates were cleaned using Piranha solution and 2 nm of Cr were then deposited, followed by 50 nm of Au, which was evaporated at ≤ 0.1 nms^{-1} and < 10^{-6} mbar using a Mantis Qprep 250 deposition system. Monolayer formation was initiated by immersing the substrate in a 1 mM solution of 11-Mercapto-1-undecanol in ethanol. The prepared SAMs were then thoroughly rinsed with ethanol and Milli-Q water. Hydroxyl-functionalized SAM surfaces were used here to minimize sample drift during the coherent imaging experiment.

Calcium Carbonate Precipitation. Calcite crystals were precipitated using the ammonia diffusion method.[1] 200 μL droplets of aqueous 5 mM $CaCl_2$ solution containing either 2 mM $MgCl_2$ or 2 mM lysine were deposited on hydroxyl-terminated gold surfaces.[2] These substrates were then placed in a sealed container (1 L) in the presence of solid $(NH_4)_2CO_3$ (2 g). The

atmospheric decomposition of $(NH_4)_2CO_3$ into gaseous CO_2 and NH_3 creates the required supersaturation for $CaCO_3$ precipitation. Calcite crystals of sizes 1-4 μm formed in approximately 30 minutes. The sample was then washed first with water and subsequently with ethanol to remove any adsorbed impurities, before being left to dry. The reaction was also scaled-up to volumes of 50 mL to facilitate analysis using techniques including atomic absorption spectroscopy (AA) and powder-XRD, and to determine the level of lysine incorporation. Precipitates were isolated by centrifugation when they had reached comparable sizes to those used in the CDI experiments.

General Sample Characterization. Scanning electron micrographs of the crystals were obtained using an FEI Nova NanoSEM 650. Primary phase identification of individual crystals was carried out by Raman spectroscopy (Renishaw Invia Raman Microscope, 785nm diode laser as excitation source, focused onto the sample using a 50x (NA ¼ 0.75) objective). The amount of magnesium in the crystals was also determined using atomic absorption (Perkin Elmer AA Analyst 400, operating with an oxy/acetylene flame), where the samples were dissolved in 4 mL of 5% nitric acid prior to analysis. The amount of lysine in the crystals was determined using a fluorescent derivation method, as outlined by Brueckner *et al.*[3] and described in detail in a previous publication.[4] Crystal polymorph and structure were determined via P-XRD.

Synchrotron PXRD and Diffraction Pattern Analysis. High-resolution P-XRD was carried out at beamline I11 at Diamond Light Source. Instrument calibration and wavelength refinement were performed with silicon standard samples from the National Bureau of Standards and Technology (NIST; Gaithersburg, MD, USA). All crystal samples were loaded into 0.7 mm borosilicate glass capillaries. A total angular range of $2\theta = 3–150°$ was collected

with a scan time of 30 minutes and samples were rotated during measurements at a rate of 1 rpm. The energy of the beamline was set to 15 keV (λ=0.824820(10)Å) for all experiments, and diffraction patterns were acquired using the beamline integrated multi-analysing crystals (MAC) detector (x5 MAC-arms with 45 MAC channels). This results in narrow diffraction peaks with an instrumental contribution to peak widths of less than 0.004°. Pattern analysis to obtain the domain sizes and strain was carried out by Rietveld analysis using the PANalytical X'Pert HighScore Plus software package.[5]

Atomic Force Microscopy (AFM). AFM measurements were carried out using a MultiMode 8 scanning probe microscope with a NanoScope V controller (Bruker). All images were obtained in contact mode using SNL-10 probes (Bruker). Calcite seed crystals were created by mixing equal volumes of 10 mM $CaCl_2$ and 10 mM $NaHCO_3$ stock solutions in the presence of a glass slide that was pre-cleaned with piranha solution. After one day, the glass slide was rinsed with ethanol, and was then dried and glued to an AFM stub. An appropriate seed crystal on this slide was selected for AFM measurements. All images were taken *in situ* using freshly prepared 0.25 mM "$CaCO_3$"/ 0.1 mM Lys/ Mg solutions. The "$CaCO_3$" component of the solution was prepared by mixing freshly prepared $CaCl_2$ and Na_2CO_3 stock solutions. Images were acquired at a scan rate of 4 Hz.

Solid-State NMR Spectroscopy. $CaCO_3$ samples enriched in ^{17}O were obtained by preparing a 1 M solution of sodium carbonate in ^{17}O water (10%) and transferring it to a sealed vessel fitted with 3 ports which were used for (a) a pH readout, and the addition of (b) 2M HCl and (c) 2M NaOH solution. Alternate additions of HCl and NaOH were made to repeatedly cycle (5 rounds) between pH levels of 4 and 10. This causes the carbonate to undergo a transition from CO_3^{2-} to CO_2, which gradually enriches the carbonate ions in the solution with ^{17}O.

Following this procedure the solution was finally adjusted to a pH of 9.5 and diluted to 20 mM. Calcite crystals were then obtained by the direct mixing of 5 mM $CaCl_2$ solution containing either 2 mM $MgCl_2$ or 2 mM lysine with 20 mM of the enriched $NaCO_3$ solution, and the crystals were isolated and dried as described above. ^{17}O enrichment levels of 5-8 mol% are expected.

Solid-state MAS NMR experiments were performed using a Bruker Avance III spectrometer equipped with a wide-bore 20.0 T magnet, operating at a ^{17}O Larmor frequency of 115.26 MHz. The samples were packed in 2.5 mm zirconia rotors and rotated at an MAS rates of 29.5 kHz. A recycle interval of 6 seconds was used between each transient. Spectra were acquired using a spin echo sequence, with a prior double frequency sweep (DFS) or hyperbolic secant pulses (HS) for signal enhancement. The DFS was applied for 800 to 300 kHz in 2 ms and the HS pulse had a ± 200 kHz offset and lasted for 2 ms. Chemical shifts are shown in ppm relative to H_2O recorded at 298 K.

Periodic density functional theory (DFT) calculations were performed using CASTEP (version 8).[6] Calculations were performed using the PBE[7] exchange correlation function with core-valence interactions described by ultra-soft pseudopotentials.[8] The first Brillioun zone was sampled using a Monkhorst–Pack grid[9] with a k-point spacing of 0.06 2π Å^{-1}. A plane wave cut-off energy of 60 Ry was applied. The initial structural models were obtained from literature and optimised prior to NMR parameters calculations allowing all atomic coordinates and cell parameters to vary. NMR parameters were then computed using the gauge-including projector augmented wave (GIPAW)[10] approach to reconstruct the all-electron wavefunction in the presence of a magnetic field. Calculations were performed on a cluster at the University of St Andrews, consisting of 300 12-core Intel Westmere nodes, connected with QDR Infiniband.

The isotropic shielding σ_{iso} is calculated from the principal components of the shielding tensor σ by $\sigma_{iso} = (\sigma_{11} + \sigma_{22} + \sigma_{33})/3$). In order to readily compare computed data with the corresponding experimental spectra it is necessary to calculate the chemical shift (δ_{iso}) by choosing a suitable reference. Computed chemical shifts were calculated using

$$\delta_{iso} = (\sigma_{ref} - \sigma_{iso}) \quad ,$$

where δ_{iso} is the computed chemical shift of the nucleus of interest, σ_{iso} is the computed chemical shielding of nucleus of interest and σ_{ref} is calculated chemical shielding of the same nucleus in a reference material. The reference material used in this case is MgO.

CDI Setup. BCDI experiments were performed at beamline 34-ID-C of the Advanced Photon Source (APS), Argonne National Laboratory USA. An undulator produced X-rays that were monochromatized using a silicon (111) double-crystal monochromator to an energy of 9 keV. Calcite crystals on a substrate were placed on a diffractometer that had its rotation centre aligned with the X-ray beam. Slits were used to aperture the X-rays to reduce the illuminated area. An X-ray sensitive charge-coupled device (Princeton instruments) with 1300 x 1300 square pixels of side length 22.5 µm was positioned at the desired diffraction angle for an off-specular (104) reflection 2.5 m from the sample. To measure 3D diffraction patterns, the crystal was rocked by 0.3^{o} through the selected reflection, with a step size of 0.003^{o} degrees. At each angle, a two-dimensional slice of the 3D far-field diffraction pattern was recorded. By stacking all two-dimensional diffraction frames together, a complete 3D diffraction pattern is obtained (Figure S2), from which real-space images can be reconstructed. Due to the small size of the crystals, the illumination can be considered to be almost coherent. Partial coherence was taken into account[11] with only a small departure from full coherence observed. To ensure that an isolated crystal was imaged, a local search in reciprocal space was performed so there was little chance of another different crystal overlapping the original crystal diffraction.

Reconstruction Algorithm. Images were obtained by performing iterative phase retrieval[12] on three-dimensional coherent diffraction patterns. Knowledge of both amplitude and phase of the diffracted wavefield allows an image to be obtained via an inverse Fourier transform. Provided the diffraction data is oversampled, that is the sample has its Fourier transform sampled at least twice the Nyquist frequency and the crystal is isolated, phase retrieval can be performed. The basic phase retrieval process begins with a guess for the diffracted phase before applying an inverse Fourier transform to yield a first estimate of the crystal. After enforcing the constraint that the crystal is isolated, this new crystal iterate is Fourier transformed to yield an estimate for the three-dimensional diffracted wave field. Consistency with the measured intensity is enforced while retaining the current estimate of the phase. This process is repeated until a self-consistent solution is reached using combinations of current and previous estimates for the crystal. For this work an approach was used which combined guided phase retrieval[13] with low to high resolution (or multi-resolution) reconstructions.[14] A detailed description can be found in reference[15]. Resolution of the final reconstructions (i-iii) was determined by comparison of the phase retrieval transfer function (PRTF) to a selected noise threshold, here e^{-1}, to be ≈95 nm (i), ≈ 130 nm (ii) and ≈ 160 nm (iii) Figure S3.[16] The PRTF is used to assess the reproducibility of the retrieved phases and hence the resolution at which features are dependably replicated.

2. Figures

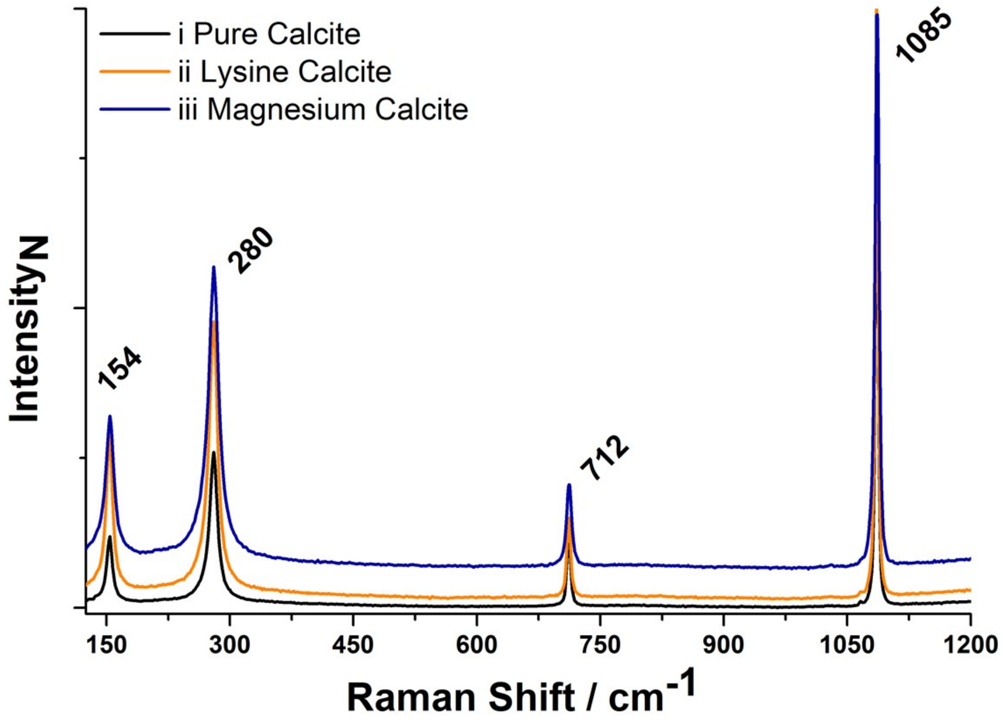

Figure S1. **Raman spectra of calcite samples (i-iii).** Normalized Raman spectra of calcium carbonate precipitates obtained via the ammonia diffusion method from 5 mM $CaCl_2$ solutions. (i) Pure calcite crystals, (ii) calcite crystals containing Lysine and (iii) calcite crystals containing magnesium. The calcite polymorph was identified based on characteristic peaks at 1085, 711, 281 and 155 cm^{-1}.[17]

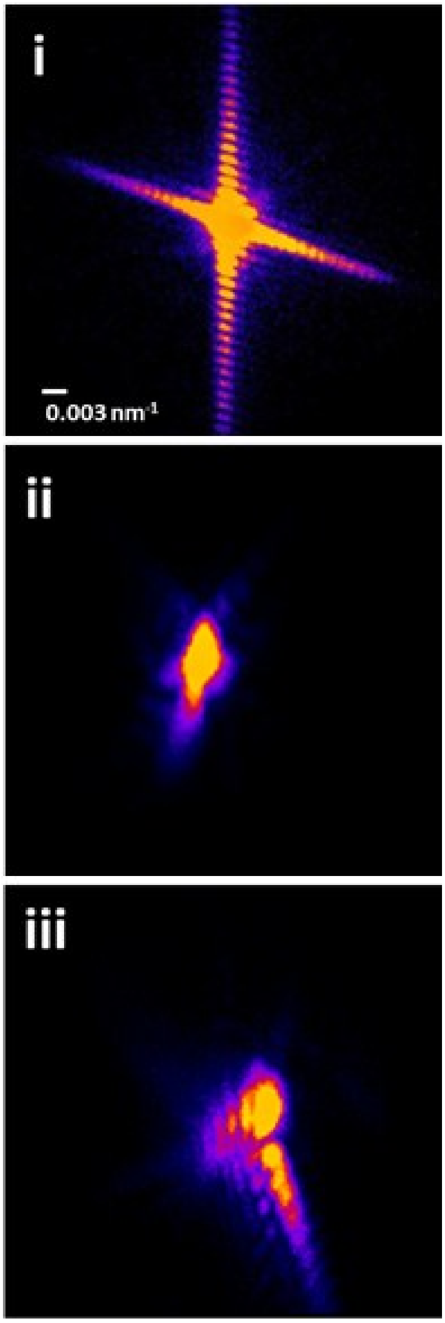

Figure S2. **CDI diffraction pattern.** Slices through the centre of the diffraction data of (i) pure calcite crystals, (ii) calcite crystals containing Lysine and (iii) calcite crystals containing magnesium. The scale is the same for all three images.

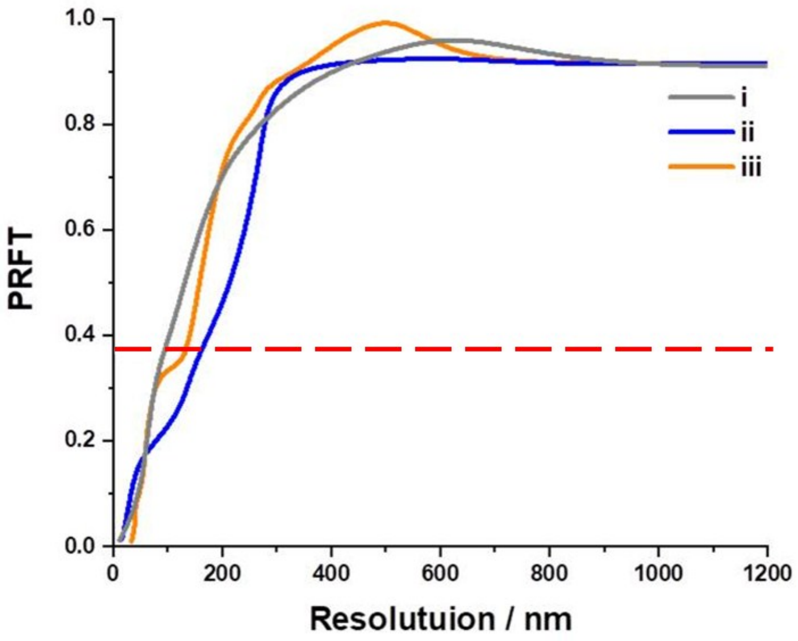

Figure S3. **Amplitude Image Resolution.** The phase retrieval transfer functions (PRTF) for the data sets from (i) pure calcite crystals, (ii) calcite crystals containing Lysine and (iii) calcite crystals containing magnesium. The PRTF measures the reproducibility of the retrieved phases. The resolution is given by the intersection of the PRTF with the selected threshold of e^{-1} or 0.37 (red line). Such selection results in conservative real-space resolution estimates of (i) ≈ 95 nm, (ii) ≈ 130 nm and (iii) ≈ 160 nm.

3. Supporting Movies 1-3

These movies provide rocking curves of the diffraction patterns collected, resulting stacked 3D diffraction pattern, and the reconstructed electron density, projected displacement and iso-surface renderings of the defects present within crystals (i-iii). The defects are highlighted in a transparent projection of the electron density. S1, S2 and S3 show the crystals (i), (ii) and (iii) which were discussed in the main text.

4. References

1. J. Ihli, P. Bots, A. N. Kulak, L. G. Benning and F. C. Meldrum, *Adv. Func. Mater.*, 2013, **23**, 1965-1973.
2. C. J. Stephens, Y.-Y. Kim, S. D. Evans, F. C. Meldrum and H. K. Christenson, *J. Am. Chem. Soc.*, 2011, **133**, 5210-5213.
3. H. Brückner, S. Haasmann, M. Langer, T. Westhauser, R. Wittner and H. Godel, *J. Chrom. A*, 1994, **666**, 259-273.
4. D. C. Green, J. Ihli, Y. Y. Kim, S. Y. Chong, P. A. Lee, C. J. Empson and F. C. Meldrum, *Cryst. Growth Des.*, 2016, **16**, 5174-5183.
5. Y. Y. Kim, J. D. Carloni, B. Demarchi, D. Sparks, D. G. Reid, M. E. Kunitake, C. C. Tang, M. J. Duer, C. L. Freeman, B. Pokroy, K. Penkman, J. H. Harding, L. A. Estroff, S. P. Baker and F. C. Meldrum, *Nature Mater.*, 2016, **15**, 903-912.
6. S. J. Clark, M. D. Segall, C. J. Pickard, P. J. Hasnip, M. J. Probert, K. Refson and M. C. Payne, *Z. Kristal.*, 2005, **220**, 567-570.
7. J. P. Perdew, K. Burke and M. Ernzerhof, *Phys. Rev. Letts.*, 1996, **77**, 3865-3868.
8. J. R. Yates, C. J. Pickard and F. Mauri, *Phys. Rev. B*, 2007, **76**, 024401.
9. H. J. Monkhorst and J. D. Pack, *Phys. Rev. B*, 1976, **13**, 5188-5192.
10. C. J. Pickard and F. Mauri, *Phys. Rev. B*, 2001, **63**, 245101.
11. J. N. Clark, X. Huang, R. Harder and I. K. Robinson, *Nature Commun.*, 2012, **3**, 993.
12. J. R. Fienup, *Appl. Opt.*, 1982, **21**, 2758-2769.
13. C.-C. Chen, J. W. Miao, C. W. Wang and T. K. Lee, *Phys. Rev. B*, 2007, **76**, 064113.
14. B. C. McCallum and R. H. T. Bates, *J Mod Opt*, 1989, **36**, 619-648.
15. J. N. Clark, J. Ihli, A. S. Schenk, Y. Y. Kim, A. N. Kulak, J. M. Campbell, G. Nisbet, F. C. Meldrum and I. K. Robinson, *Nature Mater.*, 2015, **14**, 780-784.
16. P. Thibault, V. Elser, C. Jacobsen, D. Shapiro and D. Sayre, *Acta Crystal. A*, 2006, **62**, 248-261.
17. C. G. Kontoyannis and N. V. Vagenas, *Analyst*, 2000, **125**, 251-255.
